# Survey data of Gen Z customer behaviour using food delivery applications in Vietnam

**DOI:** 10.1016/j.dib.2023.109779

**Published:** 2023-11-07

**Authors:** Tuan Duong Vu, Hoang Viet Nguyen, Phuong Thao Vu, Thi Hoang Ha Tran, Hoang Nam Nguyen, The Son Ngo

**Affiliations:** a Institute of Business Administration, Thuongmai University, Hanoi 100000, Vietnam; b Board of Rectors, Thuongmai University, Hanoi 100000, Vietnam; c Faculty of Economics and International Business, Thuongmai University, Hanoi 100000, Vietnam

**Keywords:** Food delivery applications, Diffusion of innovation, Sharing economy, Gen Z, Continuance intention, Emerging market, Vietnam

## Abstract

This study presents an analysis based on data collected via questionnaire, surveying Gen Z customers using food delivery applications in Vietnam. The purpose of the original research was to investigate factors influencing Gen Z customers' decision to continue using the applications. The data set presented in this paper includes 361 valid responses that were collected by convenience sampling method from Hanoi and Hochiminh City, which are the two most potential regions of e-commerce transactions in Vietnam. After being collected, sorted, and filtered, the data was calculated by SPSS 22 and AMOS 23 software to extract descriptive analysis, Cronbach's Alpha, and confirmatory factor analysis (CFA). The calculation results indicated that this data set ensures reliability, convergent, and discriminant validity, which can serve as a good reference for future studies.

Specifications Table

**Table:** 

Subject	Business, Management and decision sciences
Specific subject area	Marketing, Business administration, Innovation, Technology adoption, and Customer behaviour
Data format	Raw Analysed
Type of data	Table
Data collection	Survey Questionnaire (included in Supplementary Materials)
Data source location	Region, Asia Country, Vietnam
Data accessibility	Repository name Open Science Framework (OSF) Data identification number: https://doi.org/10.17605/OSF.IO/TEPXB Direct link to data: https://doi.org/10.17605/OSF.IO/TEPXB

## 1 Value of the Data

• The data provides advanced knowledge to understand several factors related to the Diffusion of Innovation, attitude, and continuance intention to use food delivery applications of Gen Z customers in an emerging market of Southeast Asia.
• The dataset is helpful for stakeholders of the sharing economy business model, such as enterprises, policy-makers, customers, and technology providers, to understand the perception of Gen Z customers using food delivery applications.
• The present data can provide significant information for researchers who intend to conduct studies related to customer behaviours using food delivery applications. Notably, the results of the dataset are essential for studies in comparison with other countries or different generations.
• The dataset is a valuable reference for studies on customer behaviours with services provided by sharing – economy business model.

## 2 Data Description

Vietnam, whose population is up to 100 million [1] and whose volume of apps downloaded ranked in the top 10 worldwide in 2021–2022 [2], is a potential market for developing app-based businesses. Food delivery applications are a relatively young but fast-growing industry in Vietnam. In 2022, this segment, together with shared transport service, is expected to surge by more than 60 % in 2025 [3]. Specifically, solely food delivery applications are currently assessed to be worth 1.1 billion dollars, served by some prominent brands like GrabFood, ShopeeFood, and Baemin [4].

The survey is designed to collect data about five principal dimensions of the DOI construct, including relative advantage, compatibility, complexity, trialability, Observability, and two factors representing customer behaviours of Gen Z, namely, attitude and continuance intention. The questionnaire consists of three main parts. Part 1 includes the research's introduction and instructions for respondents before answering the survey.

Part 2 contains the measurement scale statements, which are designed through a Likert 7-point scale (from 1 – Totally disagree to 7 – Totally agree). All variables were referenced by relevant previous studies. The first variable is compatibility (CPA), which includes four items adapted from Kim et al. [5], measured explicitly via compatibility with all aspects of life (CPA1), with the way respondent lives (CPA2), how well the app fits into respondent's lifestyle (CPA3), how the app suits the respondent generally (CPA4). The second variable is relative advantage (RA), which is adapted from Min et al. [6] and includes five items. Precisely, in comparison with other means of purchasing meals, RA is evaluated via how the apps enhance the quality of the order fulfillment (RA1), how well the apps allow buyers to control the purchasing (RA2), the level of convenience that the app brings to users (RA3), customer's general experience (RA4) and the possibility of saving money (RA5). The third variable is adapted from Tan & Teo [7]. This is Complexity (CPL) when respondents use the food delivery applications, which is assessed via three specific criteria, namely: the level of mental effort users have to take (CPL1), the frustrating feeling users may have (CPL2), the difficulty users have to deal with (CPL3). The fourth variable is adapted from Wang et al. [8], measuring Trialability (TRI) that users would like to experience when continue using the apps. Precisely, this variable is measured via how easily users may test the effectiveness of the apps (TRI1), the possibility users are allowed to experiment with different functions available on the apps (TRI2), and users' awareness of which context the app functions could be tested (TRI3). The fifth variable, which is the Observability of the fact that food delivery applications are used, is adapted from Wang et al. [8] and Min et al. [6]. In particular, the variable is assessed via how respondents can easily witness that surrounding people use the apps (OBS1), the visibility of the app service in their living area (OBS2), and the advantages of using the apps can be noticed promptly (OBS3). The sixth variable is Attitude (ATT), which is adapted from Nguyen et al. [9] and indeed assesses the respondents' attitude toward using food delivery applications. Specifically, the variable evaluates whether respondents consider using the apps as a smart solution for food purchasing (ATT1), whether they think utilizing the apps is an excellent move (ATT2), and whether they appreciate using the apps for food delivery (ATT3). Finally, the dependent variable is Continuance usage intention (INT), adapted from Lee et al. [10]. Specifically, the respondents' intention to continue using the applications is measured via four criteria, namely willingness to reuse the apps in the future (INT1); a continuous effort to use the apps to fulfill needs daily (INT2); planning to use the apps regularly (INT3); prompt intention for using in the very upcoming food purchasing (INT4).

Part 3 includes the questions about the demographic characteristics of participants. To be more specific, question 26 of the survey required respondents to select a gender group: male, female, or other. The next question asked respondents about the age group that they belonged to, namely 15–18, which implied the age of high school students in Vietnam; 19–22, which mainly covered undergraduate or vocational students in Vietnam; 23–25, which referred to the group of job-holders who were about one to three years after graduation; 26–28 which mainly covered the group of job-holders four to six years after college. Even though the survey was conducted in metropolises in Vietnam, and the authors predicted the age range might align with the typical educational features there, this prediction was not exact. Therefore, a demographics question related to educational level was added, including five common categories in Vietnam, namely: high school or lower level (e.g., secondary graduates); professional certificate that learners had graduated from high school and not enrolled in any college or university for a degree, acquiring this kind of certificate might allow them to become elemental labour force in factories, construction sites, and so on; university undergraduate degree for respondents who had graduated from a university; postgraduate degree for respondents who had acquired a Master or Ph.D. degree. Question 29 asked respondents to select an income level that reflected how much they may earn in total per month, with five levels. The last demographic question is about marital status, which may partly imply the purpose of their spending for personal or for a household. In addition, the targeted respondents of this survey were Gen Z individuals; therefore, the two most common types of marital status in Vietnam for the Gen Z group were included, namely, single and married. The option “other” was also added if the respondent did not fit into the two categories.

The detailed three-part survey, as described above, can be found in a file named 'Questionnaire and Coding.docx' via the Data identification number in the Specifications Table. This file is the document form of the survey that the authors had massively distributed to respondents via face-to-face interaction, as mentioned below. After the period of collecting data, all raw data, excluding the surveys missing one or some missing answers, were input into the Excel file named 'Dataset.xslx' via the Data identification number in the Specifications Table. The authors used the data set in this Excel file to make the following analysis in this paper.

To avoid common bias method errors, we applied several suggestions, such as changing the order of questions and ensuring the private information of participants [11]. Furthermore, Harman's single-factor analysis demonstrated that the single factor accounted for 28.499 % of the total variance explained (< 50%), and the common latent factor accounted for 13.69 % total variance (< 25 %). In addition, when comparing the estimated weight of the measurement model and the latent common factor model, the difference between the estimated weights did not exceed 0.2. Finally, the single factor CFA analysis results indicated that c2/df = 7.667, GFI = 0.648, TLI = 0.512, CFI = 0.553, and RMSEA = 0.136. These indices are less than the significant thresholds of goodness fit model indices requirements. Hence, the common bias method does not appear in this study [12] (Table 1).

**Table 1:** Demographic characteristics of respondents.

Coding in survey	Characteristics		Frequency	Percentage (%)
Q26	***Gender***	Male	172	47.65
		Female	189	52.35
Q27	***Age***	15-18	16	4.43
		19-22	89	24.65
		23-25	111	30.75
		26-28	145	40.17
Q28	***Education level***	High school or lesser	26	7.20
		Professional certificate	41	11.36
		College diploma	83	22.99
		University undergraduate degree	175	48.48
		Postgraduate degree	36	9.97
Q29	***Monthly income***	< 10,000,000 VND	183	50.69
		10,000,000 VND – 20,000,000 VND	102	28.25
		20,000,000 VND – 30,000,000 VND	43	11.91
		30,000,000 VND – 40,000,000 VND	25	6.93
		Above 40,000,000 VND	8	2.22
Q30	***Marital status***	Married	139	38.50
		Single	222	61.50

*Note*: 1 USD, approximately 23,000 VND during survey period.

Among 361 responses, the proportions of males and females were quite similar, with the higher belonging to the latter at 52.35 %. These respondents fell into four age groups ranging from 15 to 28, so their years of birth were from 1995 to 2008, fitting with the definition of Gen Z. The oldest age segment, 26-28, accounted for the most significant percentage of 40.17 %, followed by the second oldest one (23–25) of 30.75 %. The 19–22 group comprised nearly a quarter of the respondents, and the youngest segment share was the rest. This division is sensible because, from the age of 23 and upwards, respondents are typically job-holders with regular income, and the demand for shopping is higher and more frequent. In terms of educational level, the majority of respondents acquired a university undergraduate degree, making up 48.48 %. The second most popular qualification among surveyed customers was a college diploma, with approximately 23 %. For the other educational levels, like up to high school, professional certificate, and postgraduate degree, the percentages were relatively minor, namely 7.2 %, 11.36 %, and 9.97 %, respectively. Half of the respondents had an average monthly income of less than 10,000,000 VND (nearly 420 USD) when answering the questionnaire, followed by 28.25 % earning 10,000,000–20,000,000 VND per month (420–840 USD/month). Mainly at the age of 23-28, having just graduated from university for up to five years, these Gen Z respondents mainly held freshman to junior positions in their organization. For the marital status characteristics, nearly two-thirds of the respondents were single, and the rest were married.

Afterward, all survey data were analyzed through SPSS 22 and AMOS 23. These two popular software were used to test the reliability of the measurement scale and evaluate the suitability of the measurement model. Furthermore, the CB SEM also contributed to evaluating goodness fit indices, such as GFI, TLI, and CFI, and is suitable for studies with samples larger than 200 [13]. In this study, SPSS 22 software was used to test the Cronbach Alpha reliability coefficient, exploratory factor analysis (EFA), Harman's single factor test, and descriptive analysis. AMOS 23 software supports confirmatory factor analysis (CFA), common latent factor analysis, and single factor analysis.

Cronbach's Alpha test and exploratory factor analysis (EFA) were employed to test the reliability of the measurement scale. The results indicate that all Cronbach's Alpha values are ranging from 0.780 to 0.861 (> 0.7). In the EFA test, Kaiser-Meyer-Olkin (KMO) is 0.879, Sig of Bartlett's test is 0.000, the total variance explained value is 70.495 % (> 50 %), and all Eigenvalues of all factors are greater than 1.000 (Table 3).

**Table 3:** Confirmatory factor analysis (CFA), exploratory factor analysis (EFA) and Cronbach's alpha test results.

Coding in survey	Item	Fls	CR	AVE	Factor loading in the Exploratory Factor Analysis						
					CPA	RA	CPL	TRI	OBS	ATT	INT
	***Compatibility (CPA)***										
Q1	CPA1	0.783	0.862	0.609		0.773					
Q2	CPA2	0.817				0.800					
Q3	CPA3	0.800				0.782					
Q4	CPA4	0.719				0.738					
	***Relative Advantage (RA)***										
Q5	RA1	0.774	0.856	0.543	0.787						
Q6	RA2	0.788			0.767						
Q7	RA3	0.759			0.791						
Q8	RA4	0.654			0.679						
Q9	RA5	0.702			0.634						
	***Complexity (CPL)***										
Q10	CPL1	0.832	0.852	0.657				0.881			
Q11	CPL2	0.803						0.873			
Q12	CPL3	0.797						0.854			
	***Trialability (TRI)***										
Q13	TRI1	0.761	0.808	0.584					0.761		
Q14	TRI2	0.803							0.798		
Q15	TRI3	0.727							0.748		
	***Observability (OBS)***										
Q16	OBS1	0.760	0.781	0.543							0.825
Q17	OBS2	0.734									0.800
Q18	OBS3	0.717									0.833
	***Attitude (ATT)***										
Q19	ATT1	0.791	0.805	0.580						0.794	
Q20	ATT2	0.799								0.843	
Q21	ATT3	0.690								0.717	
	***Continuance intention (INT)***										
Q22	INT1	0.730	0.836	0.560			0.705				
Q23	INT2	0.708					0.696				
Q24	INT3	0.767					0.775				
Q25	INT4	0.787					0.690				
***Eigenvalues***					7.667	2.447	1.942	1.775	1.485	1.202	1.006
***Cronbach's Alpha coefficient***					0.861	0.854	0.849	0.804	0.780	0.799	0.835
***Total Variance Explained***					70.495 %						

*Note*: Fls – Factor loadings, CR – Composite reliability; AVE – Average variance extracted.

Confirmatory factor analysis (CFA) is performed through AMOS 23 software to assess the convergent and discriminant validity of the measurement scale. In this stage, goodness fit indices such as c2/df = 1.686 (< 3), GFI = 0.915 (> 0.9), TLI = 0.950 (> 0.9), CFI = 0.958, NFI = 0.903 (> 0.9) and RMSEA = 0.044 (< 0.08). In addition, Average Variance Extracted (AVE) values are greater than 0.5, Composite reliability values are greater than 0.7, and all factor loadings (Fls) are more significant than 0.6 (Table 3). Thus, convergent validity is guaranteed [13].

To check discriminant validity, we computed square root AVE and compared it with the correlation coefficients of each variable [14]. The results in Table 4 illustrated that all square root AVE values are greater than the highest correlation value of variables. Hence, discriminant validity is also ensured [13].

**Table 4:** Discriminant validity and correlation result.

	(1)	(2)	(3)	(4)	(5)	(6)	(7)
(1) Trialability	**0.764**						
(2) Relative advantage	0.612	**0.737**					
(3) Compatibility	0.563	0.559	**0.781**				
(4) Intention	0.585	0.613	0.627	**0.749**			
(5) Complexity	0.124	0.077	0.180	0.248	**0.811**		
(6) Attitude	0.426	0.425	0.408	0.601	-0.047	**0.762**	
(7) Observability	0.196	0.257	0.272	0.219	0.021	0.299	**0.737**

*Note*: Diagonal values - that are highlighted indicate the square root of AVE of the construct.

The results of descriptive statistics show that the mean of variables ranges from 3.233 to 5.114, and the standard deviation ranges from 0.787 to 1.146. In general, items with relative advantage have the highest mean among attributes, with mean values ranging from 4.701 to 5.114. On the contrary, the mean of items of complexity is significantly lower than that of the remaining items, ranging from 3.233 to 3.349. The results of the mean and standard deviation are described in Table 2.

**Table 2:** Measurement scale and descriptive analysis results.

Variable statement	Mean	SD
***Compatibility (CPA) – Adapted from Kim et al.****[**5**]*		
CPA1: Using the food delivery applications is compatible with all aspects of my life	4.654	0.931
CPA2: Using the food delivery applications fits well with the way I live	4.751	0.927
CPA3: Using the food delivery applications fits into my lifestyle	4.742	0.921
CPA4: Using the food delivery applications suits me	4.753	0.880
***Relative advantage (RA) – Adapted from Min et al.****[**6**]*		
RA1: Compared to other ways, food delivery applications improve the quality of my task	5.114	1.070
RA2: Compared to other ways, food delivery applications give me greater control over my task	4.875	1.045
RA3: Compared to other ways, food delivery applications make it more convenient	4.720	1.044
RA4: Compared to other ways, food delivery applications enhance my overall experience	4.701	0.912
RA5: Food delivery applications save me money compared to other way	4.878	0.970
***Complexity (CPL) – Adapted from Tan & Teo****[**7**]*		
CPL1: Using food delivery applications requires a lot of mental effort	3.244	0.854
CPL2: Using food delivery applications can be frustrating	3.349	0.803
CPL3: Food delivery applications are easy ways to deliver food that I ordered*	3.233	0.929
***Trialability (TRI) – Adapted from Wang et al.****[**8**]*		
TRI1: I feel it is easy to try out using food delivery applications	4.529	1.038
TRI2: Food delivery applications are open to me adequately to allow me test various functions it offers	4.626	1.033
TRI3: I know situations that I can go to try out various functions of food delivery applications	4.784	1.146
***Observability (OBS) – Adapted from Wang et al.****[**8**]****and Min et al.****[**6**]*		
OBS1: In my local area, people can easily see food delivery applications used by everyone	4.515	0.879
OBS2: Food delivery applications service activities are easily visible in my local area	4.576	0.837
OBS3: I can see the benefits of using food delivery applications immediately	4.598	0.787
***Attitude (ATT) – Adapted from Nguyen et al.****[**9**]*		
ATT1: Using food delivery applications is a smart solution	4.421	0.997
ATT2: Using food delivery applications is a good idea	4.485	0.928
ATT3: I really enjoy using food delivery applications	4.609	1.019
***Continuance usage intention (INT) – Adapted from Lee et al.****[**10**]*		
INT1: I intend to continue using food delivery applications in the future	4.698	0.840
INT2: I will always try to use food delivery applications in my daily life	4.648	0.928
INT3: I plan to continue to use food delivery applications frequently	4.709	0.901
INT4: I have decided to use food delivery applications for purchasing foods the next time	4.823	0.889

*Note*:

⁎ Reverse in coding, SD – Standard deviation.

## 3 Experimental Design, Materials and Methods

Based on previous relevant studies, the authors synthesized validated variables and items to preliminary form the measurement scale, and verbal expressions were adjusted to fit the Vietnamese communication context. Two English teaching professionals were involved in translating the scale into Vietnamese for the main part of the questionnaire. The two experts also did a crosscheck to ensure consistency and clarity according to recommendations by Harkness et al. [15]. In addition to the core segment of questions revolving around the research model, the authors added demographic questions at the end of the questionnaire. Then, the authors conducted a pre-test to ensure the appropriateness of the scale. Firstly, five experts, including two Marketing Ph.D., one food delivery application expert, and two managers working for food delivery application enterprises, participated in individual in-depth interviews to comment on the questions. Then, twenty regular users of food delivery applications took part in individual structured interviews for a second review of all statements. In interviews with expert subjects, the authors set appointments at a reasonable time when respondents were not under time pressure and felt most comfortable participating. Questions range from general information about the online food delivery service market to content related to the research topic, such as questionnaires and scales. To avoid missing any information, the authors recorded the content of the interviews. The main information was collected by extracting critical points from the interview transcript. For interviews with customers using online food delivery services, the authors encouraged customers to carefully review the content and give objective opinions on the questionnaire, such as accuracy of statements, meaning expression, and additional information based on their perspectives on this research. Considering all comments of both the experts and the customers, the authors made necessary revisions before pilot testing in a sample of 40 random Gen Z customers. Based on the results, the authors finalized a scale consisting of seven factors with 25 items whose Cronbach's Alpha coefficients for groups of variables were above 0.7 (Table 2).

After the pre-test, the questionnaire was massively sent to Gen Z customers born from 1995–2010 [16] in two metropolises in Vietnam: Hanoi and Hochiminh City, which had the highest e-commerce indexes in 2022. The two locations are the most densely populated in the country, with clusters of various businesses and academic institutions; therefore, they are the main markets for e-commerce activities in general and food delivery application services in specific [1,17]. These two metropolises are the most densely populated in Vietnam [18], holding top e-commerce indicators [19], and residents there are well familiar with food delivery applications. Hence, the two cities are suitable places to conduct surveys on behaviour when using food delivery applications. Since determining the overall sample size for this study was unavailable, this study adopted a convenience sampling strategy. Although convenience sampling strategies have poorer generalizability than probability sampling methods, this method saves costs and time for researchers. In particular, this study focused on surveying the Gen Z customer group to comply with recommendations on the homogeneous convenience sampling method, which can significantly limit the disadvantages of the convenience sampling method [20]. Finally, the convenience sampling strategy helped this study collect primary data from respondents in a short period, which was helpful in the context where food delivery application service and the business environment have rapidly changed. The authors approached potential subjects face-to-face at shopping malls, universities, and other public places to persuade them to participate in the survey. The target respondents are Gen Z customers who have used food delivery applications regularly and have good knowledge of food delivery applications. After the respondents finished the questionnaire, the authors gave them a gift worth 40,000 VND (approximately 2 USD) as an incentive.

## Limitations

Not applicable

## Ethics Statement

All subjects gave their informed consent for inclusion before they participated in the study. The study was conducted following the Declaration of Helsinki.

## CRediT authorship contribution statement

**Tuan Duong Vu:** Conceptualization, Methodology, Investigation, Formal analysis, Writing – original draft. **Hoang Viet Nguyen:** Validation, Data curation, Supervision, Writing – review & editing. **Phuong Thao Vu:** Conceptualization, Methodology, Supervision, Writing – original draft. **Thi Hoang Ha Tran:** Supervision. **Hoang Nam Nguyen:** Data curation, Supervision. **The Son Ngo:** Data curation.

## Data Availability

Survey data of Gen Z customer behaviour using food delivery applications in Vietnam (Original data) (OSFHOME)
